# (μ-Methylenediphosphonato-κ^4^ *O*,*O*′:*O*′′,*O*′′′)bis[(ethyl­ene­diamine-κ^2^ *N*,*N*′)palladium(II)] tetra­hydrate

**DOI:** 10.1107/S2056989018016419

**Published:** 2018-11-22

**Authors:** Viktoriya V. Dyakonenko, Alexandra N. Kozachkova, Natalia V. Tsaryk, Vasily I. Pekhnyo, Ruslan V. Lavryk

**Affiliations:** a SSI "Institute for Single Crystals", National Academy of Sciences of Ukraine, Nauki Ave 60, Kharkiv 61001, Ukraine; b V.I. Vernadsky Institute of General and Inorganic Chemistry of the National Academy of Sciences of Ukraine, Kyiv, Ukraine; c National University of Life and Environmental Science of Ukraine, Kyiv, Ukraine

**Keywords:** crystal structure, ethyl­enedi­amine palladium, methyl­enedi­phospho­nate, anti­cancer agents

## Abstract

The title compound, [Pd_2_(C_2_H_8_N_2_)_2_(CH_2_O_6_P_2_)]·4H_2_O, comprises of a binuclear mol­ecule (point group symmetry 2), with a twofold rotation axis running through the central C atom of the methyl­enedi­phospho­nate (MDP) anion. The Pd^II^ atom has a square-planar coordination environment defined by the N atoms of a bidentate ethyl­enedi­amine (en) ligand and two O atoms of the bridging MDP anion. In the crystal structure, metal complexes are arranged in layers parallel (001) and are sandwiched between layers containing disordered water mol­ecules of crystallization. Extensive intra­layer hydrogen bonds of the type N—H⋯O in the metal complex layer and O—H⋯O in the water layer, as well as O—H⋯O hydrogen bonds between the two types of layers, lead to the formation a three-dimensional network structure. The two lattice water mol­ecules are each equally disordered over two positions.

## Abstract 2

The binuclear title mol­ecule exhibits point group symmetry 2, with the Pd^II^ atom in a square-planar coordination environment defined by two N atoms from ethyl­enedi­amine and two O atoms from methyl­enedi­phospho­nate ligands.

## Chemical context   

Platinum drugs are some of the most important and clinically applied anti-cancer agents. Di­phospho­nic acids are therapeutic agents for treating osteoporosis and metastatic bone diseases. Therefore new complexes designed with a combin­ation of platinum group metals with di­phospho­nic acid (or derivatives thereof) as bone-targeting groups can improve the chemotherapeutic efficacy in the treatment of bone cancer and can reduce adverse effects. Methyl­enedi­phospho­nic acid (medronic acid, MDP, H_4_ *L*) is the smallest bis­phospho­nate, which accumulates on the sites of osteoid mineralization and can be used in combination with platinum metals to treat cancer and metastatic bone diseases. Platinum–bis­phospho­nate complexes, including bis­{ethyl­enedi­amine)­platinum(II)}medronate, as novel Pt-prodrugs in the local treatment of bone tumors have been reported (Wani *et al.*, 2016 ▸; Iafisco *et al.*, 2009 ▸; Palazzo *et al.*, 2007 ▸; Iafisco & Margiotta, 2012 ▸; Margiotta *et al.*, 2009 ▸). The preparation and structure determination of [Pt_2_(cis-di­amino­hexa­ne)_2_(methyl­enedi­phospho­n­ate)] was reported by Bau *et al.* (1988 ▸).
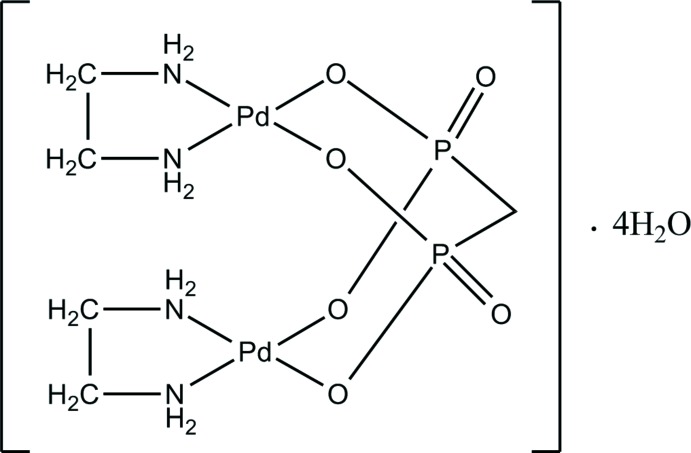


In this work, we synthesized a new platinum metal complex, *viz*. [Pd_2_(C_2_H_8_N_2_)_2_(CH_2_O_6_P_2_)]·4H_2_O or [Pd_2_(en)_2_(MDP)]·4H_2_O, and report here its mol­ecular and crystal structures.

## Structural commentary   

**Figure 1:**
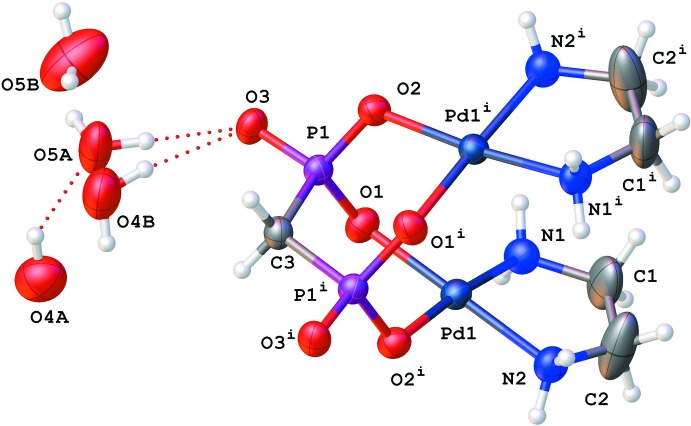
Mol­ecular structure of the title complex and surrounding water mol­ecules with displacement ellipsoids drawn at the 50% probability level. [Symmetry code: (i) −*x* + 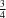, −*y* + 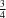, *z*.]

The binuclear [Pd_2_(en)_2_(MDP)] complex mol­ecule is uncharged and exhibits point group symmetry 2, with the twofold rotation axis passing through the central C atom of the MDP ligand (Fig. 1 ▸). The Pd^II^ atom has a square-planar environment defined by two nitro­gen atoms (N1 and N2) of the chelating en ligand and two oxygen atoms (O2 and O3) of the bis-bidentately coordinating MDP ligand that bridges two symmetry-related Pd^II^ atoms into the binuclear complex. The deviation of the Pd1 site from the least-squares plane involving the ligand atoms N1, N2, O2 and O3 is 0.06 Å. The Pd—N and Pd—O bond lengths are in typical ranges whereby the Pd—N bonds are about 0.02 Å shorter than the Pd—O bonds (Table 1 ▸). The O—Pd—O, N—Pd—N and O—Pd—N bond angles vary within 84.5 (2)°–93.3 (2)° (Table 1 ▸) and indicate a slight distortion from a square-planar coordination. In general, the structural features of the [Pd_2_(en)_2_(MDP)] complex are similar to those of the related complex [Pd_2_(en)_2_EDP] where EDP is 1-hy­droxy­ethane 1,1-di­phospho­nate or etidronate (Kozachkova *et al.*, 2018 ▸).

The Pd1—O1—P1—C3—P1^i^—O1^i^ [symmetry code: (i) 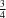 − *x*, 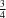 − *y*, *z*] six-membered metallacycle adopts a chair conformation; the puckering parameters (Cremer & Pople, 1975 ▸; Zefirov *et al.*, 1990 ▸) are *S* = 1.19, *ψ* = 16.52°, *θ* = 3.02°. The Pd1 and C3 atoms deviate by −0.95 and 0.82 Å, respectively, from the least-squares plane of the remaining atoms of this metallacycle with an s.u. of 0.01 Å. The Pd1—N1—C2—C1—N2 five-membered metallacycle involving the en ligand adopts an envelope conformation. The N1 atom deviates by 0.27 Å from the mean plane of the remaining atoms (the s.u. of this plane is 0.005 Å).

## Supra­molecular features   

**Figure 2:**
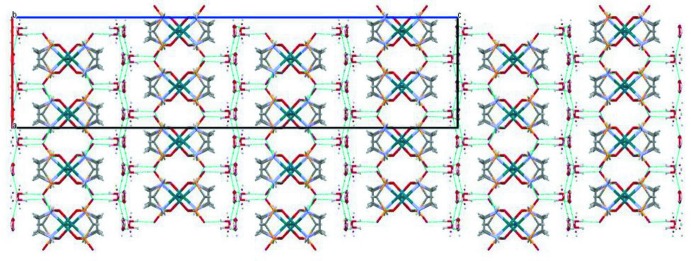
Crystal packing of the title compound in a view along [010] with hydrogen bonds shown as dashed lines.

In the crystal, [Pd_2_(en)_2_(MDP)] mol­ecules are linked via _(en)_N1—H1*A*⋯O1^ii^_(MDP)_ hydrogen bonds (Fig. 2 ▸, Table 2 ▸), forming chains parallel to [010]. Neighboring chains are connected to each other through _(en)_N2—H2*A*⋯O3^iii^_(MDP)_ and _(en)_N1—H1*B*⋯O3^iv^_(MDP)_ hydrogen bonds, forming layers parallel to (001). The disordered water mol­ecules O4 and O5 are located between these layers and are connected to each other by O—H⋯O hydrogen bonds (Table 2 ▸), forming a sandwich-type structure. The structural disorder of the water mol­ecules is probably caused by different possibilities of possible positions favourable for hydrogen bonding with neighbouring mol­ecules. Alternating layers containing [Pd_2_(en)_2_(MDP)] complexes and water mol­ecules are stacked along [001] and are linked to each other by a series of O—H⋯O hydrogen bonds (O5*A*—H5*AB*⋯O3 and O4*B*—H4*BB*⋯O3; Table 2 ▸).

## Database survey   

A search of the Cambridge Structural Database (CSD, Version 5.39, update November 2017; Groom *et al.*, 2016 ▸) for complexes containing the Pd(en) moiety yielded 226 hits with a mean Pd—N bond lengths of 2.028 Å. A search for Pd complexes with MPD as a ligand revealed only one entry (Kutsenko *et al.*, 2014 ▸) with Pd—O bond lengths of 1.999 and 2.004 Å.

## Synthesis and crystallization   

[Pd_2_(C_2_H_8_N_2_)_2_(CH_2_O_6_P_2_)]·4H_2_O was prepared as previously reported in the literature (Kozachkova *et al.*, 2018 ▸). A solution of AgNO_3_ (0.4 mmol, 0.0679 g) in water (3 ml) was added to a suspension of [Pd(en)Cl_2_] (0.2 mmol, 0.0474 g) in water (6 ml) under constant stirring and heating at 333 K for 30 min. The resulting suspension was refrigerated to facilitate the precipitation of AgCl. The solid material was removed by suction filtration. MDP (0.1 mmol, 0.0176 g) was added to the filtrate, and the pH was adjusted to 6 by addition of KOH (0.1 mol l^−1^). The obtained slightly yellow solution was heated at 333 K for 20 min and then left to evaporate at room temperature. Yellow rectangular crystals of the title compound suitable for crystallographic studies were grown by slow evaporation of an aqueous solution at room temperature.

Compound [Pd_2_(en)_2_(MDP)]·4H_2_O: yield 86%. Analysis found: C, 11.5; H, 3.6; N, 10.9; P, 11.8; Pd, 40.8. Calculated for C_5_H_26_N_4_O_6_P_2_Pd_2_: C, 11.9; H, 3.9; N, 11.1; P, 12.3; Pd, 41.9%. The ^31^P-{H} NMR spectrum of an aqueous solutions of the synthesized compound exhibited a singlet with δ^P^ 35.0 ppm.

## Refinement   

Crystal data, data collection and structure refinement details are summarized in Table 3 ▸. Hydrogen atoms of the metal complex were located from difference-Fourier syntheses. The H atom of the central CH_2_ bridge of the MDP ligand was refined freely, and the methyl­ene and NH_2_ hydrogen atoms of the en ligand were treated in the riding-model approximation with C—H = 0.97 Å (N—H = 0.89 Å) and *U*_iso_(H) = 1.2*U*_eq_(C,N). The two lattice water mol­ecules (O4 and O5) are each disordered over two positions and were refined with occupancies 0.5:0.5 each. Their hydrogen atoms were calculated, taking into account the direction of expected hydrogen bonds. The positions of these hydrogen atoms were fixed at the last steps of refinement with O—H = 0.85 Å, and with *U*_iso_(H) = 1.5*U*_eq_(O).

## Supplementary Material

Crystal structure: contains datablock(s) I. DOI: 10.1107/S2056989018016419/wm5473sup1.cif

Structure factors: contains datablock(s) I. DOI: 10.1107/S2056989018016419/wm5473Isup2.hkl

CCDC reference: 1879750

Additional supporting information:  crystallographic information; 3D view; checkCIF report

## Figures and Tables

**Table 1:** Selected geometric parameters (Å, °)

Pd1—O1	2.033 (5)	Pd1—N2	2.009 (6)
Pd1—O2^i^	2.046 (5)	Pd1—N1	2.021 (5)
			
O1—Pd1—O2^i^	93.32 (19)	N2—Pd1—N1	84.5 (2)
N2—Pd1—O2^i^	91.2 (2)	N1—Pd1—O1	90.8 (2)

Symmetry code: (i) 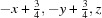.

**Table 2:** Hydrogen-bond geometry (Å, °)

*D*—H⋯*A*	*D*—H	H⋯*A*	*D*⋯*A*	*D*—H⋯*A*
N2—H2*A*⋯O3^ii^	0.89	2.07	2.932 (8)	163
N1—H1*A*⋯O1^iii^	0.89	2.21	3.006 (8)	149
N1—H1*B*⋯O3^iv^	0.89	2.06	2.906 (8)	159
O4*A*—H4*AA*⋯O5*A*	0.86	2.05	2.79 (3)	145
O5*B*—H5*BA*⋯O5*B* ^v^	0.85	1.56	2.17 (4)	127
O5*B*—H5*BA*⋯O4*B* ^v^	0.85	2.29	2.97 (3)	136
O5*B*—H5*BB*⋯O5*B* ^vi^	0.85	1.97	2.73 (4)	148
O5*B*—H5*BB*⋯O4*B* ^vii^	0.85	2.24	2.78 (2)	121
O5*A*—H5*AA*⋯O4*A* ^vii^	0.85	1.98	2.786 (19)	158
O5*A*—H5*AB*⋯O3	0.85	1.83	2.675 (16)	176
O4*B*—H4*BB*⋯O3	0.85	1.93	2.761 (18)	165

Symmetry codes: (ii) 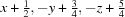; (iii) 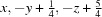; (iv) 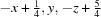; (v) 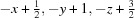; (vi) 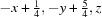; (vii) 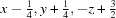.

**Table 3:** Experimental details

Crystal data	
Chemical formula	[Pd_2_(C_2_H_8_N_2_)_2_(CH_2_O_6_P_2_)]·4H_2_O
*M*_r_	577.04
Crystal system, space group	Orthorhombic, *F* *d* *d* *d*
Temperature (K)	294
*a*, *b*, *c* (Å)	11.8871 (6), 12.5405 (6), 48.052 (2)
*V* (Å^3^)	7163.2 (6)
*Z*	16
Radiation type	Mo *K*α
μ (mm^−1^)	2.24
Crystal size (mm)	0.6 × 0.4 × 0.2

Data collection	
Diffractometer	Rigaku Oxford Diffraction Xcalibur, Sapphire3
Absorption correction	Multi-scan (*CrysAlis PRO*; Rigaku OD, 2018 ▸)
*T*_min_, *T*_max_	0.418, 1.000
No. of measured, independent and observed [*I* > 2σ(*I*)] reflections	14597, 2065, 1746
*R*_int_	0.075
(sin θ/λ)_max_ (Å^−1^)	0.649

Refinement	
*R*[*F* ^2^ > 2σ(*F* ^2^)], *wR*(*F* ^2^), *S*	0.063, 0.175, 1.14
No. of reflections	2065
No. of parameters	126
H-atom treatment	H-atom parameters constrained
Δρ_max_, Δρ_min_ (e Å^−3^)	1.69, −2.06

Computer programs: *CrysAlis PRO* (Rigaku OD, 2018 ▸), *SHELXT* (Sheldrick, 2015*a* ▸), *SHELXL* (Sheldrick, 2015*b* ▸) and *OLEX2* (Dolomanov *et al.*, 2009 ▸).

## supplementary crystallographic information

### Crystal data

**Table:**

[Pd_2_(C_2_H_8_N_2_)_2_(CH_2_O_6_P_2_)]·4H_2_O	*D*_x_ = 2.140 Mg m^−^^3^
*M**_r_* = 577.04	Mo *K*α radiation, λ = 0.71073 Å
Orthorhombic, *F**d**d**d*	Cell parameters from 2899 reflections
*a* = 11.8871 (6) Å	θ = 3.2–30.1°
*b* = 12.5405 (6) Å	µ = 2.24 mm^−^^1^
*c* = 48.052 (2) Å	*T* = 294 K
*V* = 7163.2 (6) Å^3^	Block, colourless
*Z* = 16	0.6 × 0.4 × 0.2 mm
*F*(000) = 4576	

### Data collection

**Table:**

Rigaku Oxford Diffraction Xcalibur, Sapphire3 diffractometer	2065 independent reflections
Radiation source: fine-focus sealed X-ray tube, Enhance (Mo) X-ray Source	1746 reflections with *I* > 2σ(*I*)
Graphite monochromator	*R*_int_ = 0.075
Detector resolution: 16.1827 pixels mm^-1^	θ_max_ = 27.5°, θ_min_ = 3.2°
ω scans	*h* = −15→8
Absorption correction: multi-scan (*CrysAlisPro*; Rigaku OD, 2018)	*k* = −16→16
*T*_min_ = 0.418, *T*_max_ = 1.000	*l* = −58→62
14597 measured reflections	

### Refinement

**Table:**

Refinement on *F*^2^	0 restraints
Least-squares matrix: full	Hydrogen site location: mixed
*R*[*F*^2^ > 2σ(*F*^2^)] = 0.063	H-atom parameters constrained
*wR*(*F*^2^) = 0.175	*w* = 1/[σ^2^(*F*_o_^2^) + (0.0908*P*)^2^ + 141.6934*P*] where *P* = (*F*_o_^2^ + 2*F*_c_^2^)/3
*S* = 1.14	(Δ/σ)_max_ = 0.002
2065 reflections	Δρ_max_ = 1.69 e Å^−^^3^
126 parameters	Δρ_min_ = −2.06 e Å^−^^3^

### Special details

**Table:**

Geometry. All esds (except the esd in the dihedral angle between two l.s. planes) are estimated using the full covariance matrix. The cell esds are taken into account individually in the estimation of esds in distances, angles and torsion angles; correlations between esds in cell parameters are only used when they are defined by crystal symmetry. An approximate (isotropic) treatment of cell esds is used for estimating esds involving l.s. planes.

### Fractional atomic coordinates and isotropic or equivalent isotropic displacement parameters (Å^2^)

**Table:**

	*x*	*y*	*z*	*U*_iso_*/*U*_eq_	Occ. (<1)
Pd1	0.37981 (4)	0.24678 (4)	0.61611 (2)	0.0284 (2)	
P1	0.24784 (12)	0.37098 (12)	0.66205 (3)	0.0273 (4)	
O1	0.2537 (4)	0.2699 (4)	0.64405 (11)	0.0346 (10)	
O2	0.2468 (4)	0.4739 (4)	0.64479 (10)	0.0322 (10)	
O3	0.1464 (4)	0.3667 (4)	0.68076 (9)	0.0362 (10)	
N2	0.4951 (5)	0.2116 (5)	0.58694 (13)	0.0387 (13)	
H2A	0.542935	0.265756	0.585250	0.046*	
H2B	0.533949	0.154405	0.592230	0.046*	
N1	0.2672 (5)	0.2073 (5)	0.58635 (12)	0.0355 (12)	
H1A	0.235915	0.144735	0.590388	0.043*	
H1B	0.212909	0.256180	0.585660	0.043*	
C3	0.375000	0.375000	0.68243 (19)	0.0299 (17)	
H3	0.373 (6)	0.437 (6)	0.6938 (17)	0.036*	
C1	0.3239 (8)	0.2011 (11)	0.55937 (18)	0.073 (3)	
H1C	0.293176	0.140753	0.549266	0.087*	
H1D	0.305487	0.264816	0.548883	0.087*	
C2	0.4418 (8)	0.1906 (13)	0.5599 (2)	0.089 (4)	
H2C	0.473610	0.239097	0.546319	0.107*	
H2D	0.461070	0.118672	0.554208	0.107*	
O4A	0.1779 (11)	0.1374 (12)	0.7512 (3)	0.072 (4)	0.5
H4AA	0.162933	0.203516	0.754198	0.108*	0.5
H4AB	0.115953	0.105016	0.747549	0.108*	0.5
O5B	0.1617 (16)	0.5219 (16)	0.7487 (5)	0.108 (7)	0.5
H5BA	0.226607	0.542097	0.753922	0.163*	0.5
H5BB	0.118927	0.574297	0.752362	0.163*	0.5
O5A	0.1535 (13)	0.3512 (16)	0.7363 (3)	0.064 (4)	0.5
H5AA	0.084422	0.344140	0.740362	0.096*	0.5
H5AB	0.154092	0.358020	0.718632	0.096*	0.5
O4B	0.1903 (15)	0.2992 (16)	0.7344 (4)	0.069 (5)	0.5
H4BA	0.228698	0.242206	0.733009	0.104*	0.5
H4BB	0.164928	0.316206	0.718439	0.104*	0.5

### Atomic displacement parameters (Å^2^)

**Table:**

	*U*^11^	*U*^22^	*U*^33^	*U*^12^	*U*^13^	*U*^23^
Pd1	0.0273 (3)	0.0295 (4)	0.0285 (4)	0.00014 (16)	−0.00047 (18)	−0.00197 (17)
P1	0.0229 (7)	0.0320 (8)	0.0270 (8)	−0.0004 (5)	0.0012 (6)	0.0012 (6)
O1	0.028 (2)	0.041 (3)	0.035 (3)	−0.0021 (18)	0.0028 (19)	−0.005 (2)
O2	0.028 (2)	0.035 (2)	0.034 (2)	0.0019 (17)	0.0010 (18)	0.0029 (19)
O3	0.026 (2)	0.051 (3)	0.031 (2)	0.0004 (18)	0.0045 (18)	−0.001 (2)
N2	0.032 (3)	0.047 (3)	0.036 (3)	0.000 (2)	0.002 (2)	0.000 (3)
N1	0.031 (3)	0.040 (3)	0.035 (3)	−0.003 (2)	−0.006 (2)	−0.005 (2)
C3	0.028 (4)	0.036 (5)	0.025 (4)	−0.001 (3)	0.000	0.000
C1	0.061 (6)	0.126 (10)	0.031 (4)	0.010 (6)	−0.010 (4)	−0.013 (5)
C2	0.047 (5)	0.176 (13)	0.045 (5)	−0.020 (7)	0.009 (4)	−0.028 (7)
O4A	0.058 (7)	0.081 (10)	0.077 (10)	0.006 (7)	−0.006 (7)	−0.007 (8)
O5B	0.074 (11)	0.117 (15)	0.134 (17)	−0.013 (10)	0.010 (11)	−0.061 (13)
O5A	0.046 (8)	0.114 (16)	0.032 (6)	0.000 (8)	0.002 (5)	−0.002 (8)
O4B	0.064 (11)	0.097 (14)	0.047 (8)	0.001 (9)	0.009 (7)	0.002 (9)

### Geometric parameters (Å, º)

**Table:**

Pd1—Pd1^i^	3.2180 (9)	C3—H3	0.95 (8)
Pd1—Pd1^ii^	3.1715 (9)	C3—H3^i^	0.95 (8)
Pd1—O1	2.033 (5)	C1—H1C	0.9700
Pd1—O2^i^	2.046 (5)	C1—H1D	0.9700
Pd1—N2	2.009 (6)	C1—C2	1.408 (14)
Pd1—N1	2.021 (5)	C2—H2C	0.9700
P1—O1	1.536 (5)	C2—H2D	0.9700
P1—O2	1.534 (5)	O4A—H4AA	0.8600
P1—O3	1.506 (4)	O4A—H4AB	0.8590
P1—C3	1.802 (5)	O4A—H4AB^iii^	0.56 (2)
N2—H2A	0.8900	O5B—H5BA	0.8503
N2—H2B	0.8900	O5B—H5BB	0.8504
N2—C2	1.468 (11)	O5A—H5AA	0.8495
N1—H1A	0.8900	O5A—H5AB	0.8515
N1—H1B	0.8900	O4B—H4BA	0.8502
N1—C1	1.464 (11)	O4B—H4BB	0.8500
			
Pd1^ii^—Pd1—Pd1^i^	164.246 (17)	Pd1—N1—H1B	109.8
O1—Pd1—Pd1^i^	80.27 (14)	H1A—N1—H1B	108.2
O1—Pd1—Pd1^ii^	87.69 (14)	C1—N1—Pd1	109.6 (5)
O1—Pd1—O2^i^	93.32 (19)	C1—N1—H1A	109.8
O2^i^—Pd1—Pd1^i^	81.11 (13)	C1—N1—H1B	109.8
O2^i^—Pd1—Pd1^ii^	89.54 (13)	P1—C3—P1^i^	114.2 (5)
N2—Pd1—Pd1^i^	104.11 (19)	P1—C3—H3	108 (4)
N2—Pd1—Pd1^ii^	88.64 (19)	P1—C3—H3^i^	108 (4)
N2—Pd1—O1	174.2 (2)	P1^i^—C3—H3^i^	108 (4)
N2—Pd1—O2^i^	91.2 (2)	P1^i^—C3—H3	108 (4)
N2—Pd1—N1	84.5 (2)	H3—C3—H3^i^	110 (10)
N1—Pd1—Pd1^ii^	87.42 (18)	N1—C1—H1C	108.2
N1—Pd1—Pd1^i^	102.77 (18)	N1—C1—H1D	108.2
N1—Pd1—O1	90.8 (2)	H1C—C1—H1D	107.3
N1—Pd1—O2^i^	174.8 (2)	C2—C1—N1	116.5 (8)
O1—P1—C3	106.9 (2)	C2—C1—H1C	108.2
O2—P1—O1	113.0 (3)	C2—C1—H1D	108.2
O2—P1—C3	106.1 (2)	N2—C2—H2C	108.4
O3—P1—O1	110.1 (3)	N2—C2—H2D	108.4
O3—P1—O2	110.3 (3)	C1—C2—N2	115.4 (8)
O3—P1—C3	110.4 (3)	C1—C2—H2C	108.4
P1—O1—Pd1	121.6 (3)	C1—C2—H2D	108.4
P1—O2—Pd1^i^	120.7 (3)	H2C—C2—H2D	107.5
Pd1—N2—H2A	109.4	H4AA—O4A—H4AB	108.2
Pd1—N2—H2B	109.4	H4AA—O4A—H4AB^iii^	72.3
H2A—N2—H2B	108.0	H4AB—O4A—H4AB^iii^	38.4
C2—N2—Pd1	111.2 (5)	H5BA—O5B—H5BB	104.5
C2—N2—H2A	109.4	H5AA—O5A—H5AB	104.4
C2—N2—H2B	109.4	H4BA—O4B—H4BB	109.4
Pd1—N1—H1A	109.8		
			
Pd1—N2—C2—C1	1.3 (14)	O3—P1—O1—Pd1	−179.9 (3)
Pd1—N1—C1—C2	18.4 (13)	O3—P1—O2—Pd1^i^	−177.9 (3)
O1—P1—O2—Pd1^i^	−54.3 (4)	O3—P1—C3—P1^i^	179.4 (2)
O1—P1—C3—P1^i^	59.7 (2)	N1—C1—C2—N2	−13.4 (17)
O2—P1—O1—Pd1	56.3 (4)	C3—P1—O1—Pd1	−60.0 (4)
O2—P1—C3—P1^i^	−61.1 (2)	C3—P1—O2—Pd1^i^	62.5 (4)

Symmetry codes: (i) −*x*+3/4, −*y*+3/4, *z*; (ii) *x*, −*y*+1/4, −*z*+5/4; (iii) −*x*+1/4, −*y*+1/4, *z*.

### Hydrogen-bond geometry (Å, º)

**Table:**

*D*—H···*A*	*D*—H	H···*A*	*D*···*A*	*D*—H···*A*
N2—H2*A*···O3^iv^	0.89	2.07	2.932 (8)	163
N1—H1*A*···O1^ii^	0.89	2.21	3.006 (8)	149
N1—H1*B*···O3^v^	0.89	2.06	2.906 (8)	159
O4*A*—H4*AA*···O5*A*	0.86	2.05	2.79 (3)	145
O5*B*—H5*BA*···O5*B*^vi^	0.85	1.56	2.17 (4)	127
O5*B*—H5*BA*···O4*B*^vi^	0.85	2.29	2.97 (3)	136
O5*B*—H5*BB*···O5*B*^vii^	0.85	1.97	2.73 (4)	148
O5*B*—H5*BB*···O4*B*^viii^	0.85	2.24	2.78 (2)	121
O5*A*—H5*AA*···O4*A*^viii^	0.85	1.98	2.786 (19)	158
O5*A*—H5*AB*···O3	0.85	1.83	2.675 (16)	176
O4*B*—H4*BB*···O3	0.85	1.93	2.761 (18)	165

Symmetry codes: (ii) *x*, −*y*+1/4, −*z*+5/4; (iv) *x*+1/2, −*y*+3/4, −*z*+5/4; (v) −*x*+1/4, *y*, −*z*+5/4; (vi) −*x*+1/2, −*y*+1, −*z*+3/2; (vii) −*x*+1/4, −*y*+5/4, *z*; (viii) *x*−1/4, *y*+1/4, −*z*+3/2.
